# Saliva and Blood Asprosin Hormone Concentration Associated with Obesity

**DOI:** 10.1155/2019/2521096

**Published:** 2019-03-27

**Authors:** Kader Ugur, Suleyman Aydin

**Affiliations:** ^1^Department of Internal Medicine (Endocrinology and Metabolism Diseases), School of Medicine, Fırat University, 23119 Elazig, Turkey; ^2^Department of Medical Biochemistry and Clinical Biochemistry, (Firat Hormones Research Group), Medical School, Fırat University, 23119 Elazig, Turkey

## Abstract

**Background:**

The aim was to investigate the amounts of saliva and serum asprosin in order to determine whether it is related to obesity and whether salivary glands synthesize asprosin or not.

**Methods:**

A total of 116 underweight, normal weight, overweight, and obese (class I, class II, and class III) volunteers participated in the study. Saliva and blood samples were collected simultaneously from the participants. The amounts of asprosin in saliva, salivary gland tissue supernatants, and bloods were determined by ELISA, whereas asprosin synthesis sites of salivary gland tissues were determined immunohistochemically.

**Results:**

The amount of asprosin from the lowest to the highest was in the order as follows: underweight, normal weight (control), overweight, and obese classes I and III. The lowest level of asprosin was detected in underweight individuals. It was also found that the interlobular striated ducts and the interlobular ducts of the submandibular and parotid salivary glands produce asprosin. According to these data, the asprosin level is related with obesity as the amount increases in accordance with increasing body mass index (BMI). On the other hand, there is also a relationship between the underweight and asprosin because the amount decreases with BMI decrease.

**Conclusions:**

Asprosin, a new adipokine, may be a novel indicator of adipose tissue mass. Therefore, we anticipate that antiasprosin preparations may be an alternative in the treatment of obesity in the future.

## 1. Introduction

Obesity is a very serious public health problem in the world with rapidly increasing prevalence; it causes significant morbidity and mortality related to many diseases, such as diabetes, atherosclerotic vascular disease, hypertension, respiratory system diseases, digestive system diseases, cancer, joint diseases, and psychological disorders [1, 2]. According to the World Health Organization (WHO), overweight and obesity prevalence is highest in the United States of America (62% for overweight and 26% for obesity for both genders) and is lowest in South-East Asia (14% for overweight and 3% for obesity for both genders). More than 50% of women are overweight in Europe, Eastern Mediterranean, and America (23% in Europe, 24% in the Eastern Mediterranean, and 29% in America) [3], making obesity more common in women worldwide [4].

Although the etiopathology of obesity has not yet been fully elucidated, the most important implicated factors include sociocultural factors, educational level, income status, age, gender, intestinal microbiota composition, genetic factors, excessive and bad nutritional habits, inadequate physical activity, and hormonal and metabolic factors [1, 5, 6]. Obesity is a long-term process that often begins in childhood. Excessive obesity slowly develops in adulthood due to changes in metabolism, with the overall weight gain being generally between 25 and 44 years of age [7, 8].

In recent years, a large number of hormones (the appetite hormones, both orexigenic and unorexigenic) that have roles in metabolism and body fat distribution have been investigated in detail to elucidate the etiopathology of obesity [9]. According to the data of the most studied metabolism hormones, leptin and nesfatin-1 levels increase with obesity [10, 11], whereas the amount of ghrelin decreases [12]. It has been suggested that these decreasing and increasing hormones are intimately involved in the etiology of obesity either by suppressing or adjusting appetite or by acting on glucose homeostasis or fat metabolism [13].

As understood from the data briefly summarized above, there is a direct link between obesity development and hormones that affect metabolism and glucose homoeostasis [14]. The newly discovered asprosin, discussed below in relation to the functions and diseases and with an effect on glucose metabolism, may be related to obesity. Asprosin was discovered by Romere et al. [15]. It is a hormone derived from adipokine, cleaved from the C-terminal portion of profibrillin. This hormone is regulated by fasting, can be found in nanomolar levels in the blood, and causes rapid release of hepatic glucose by activating the G protein-cAMP-PKA pathway in the liver [15–20]. The level of asprosin increases in insulin-resistant animals and humans [16]. Furthermore, asprosin levels increase as glucose levels decrease and vice versa (for example, with nutrition) [16]. Additionally, there is a positive correlation between plasma asprosin levels and waist circumference (WC), fasting plasma glucose (FPG), postchallenge plasma glucose (2hPG), HbA1c, triglyceride (TG), and insulin resistance (HOMA-IR) [16]. On the basis of the data, these investigators have suggested that circulating asprosin may be a marker for early diagnosis in diabetes mellitus.

In the light of all this information, we show for the first time how the amount of asprosin changes in blood and saliva of underweight (thin), normal weight, and overweight individuals and participants with classes I, II, and III obesity and also whether or not salivary glands synthesize asprosin.

## 2. Material and Method

This study was started with the permission of the noninvasive local ethics committee of the Faculty of Medicine of Fırat University, the decision dated 5.7.2018 and numbered 03. This study was conducted with the collaboration of the Endocrinology and Metabolic Diseases Clinic and Medical Biochemistry Department. We have complied with the World Medical Association Declaration of Helsinki regarding ethical conduct of research involving human subjects.

Formation of the groups in this study was based on the Canadian Guidelines for Body Weight Classification in Adults, 2003 (underweight: <18.5, normal weight: 18.5–24.9, overweight: 25.0-29.9, and obese (class I: 30.0-34.9, class II: 35.0-39.9, and class III: ≥40.0)). BMIs were calculated by dividing the participants' weights (kg) by the square of their height (m^2^) (i.e., BMI: kg/m^2^) [21]. A visual analog scale (VAS) was also used to assess participants' appetite. Patients with similar VAS were included [22].

A total of 116 volunteers, including 8 of low weight, 44 of normal weight, 19 of overweight, 10 of class I, 13 of class II, and 22 of class III, participated. Five mL venous blood and 2 mL saliva were collected concurrently following overnight fasting of the participants, as previously described [23]. Patients with acute infection, those with chronic medical illnesses, those with type 1 or type 2 diabetes (including family history), those with moderate to severe hypertension (resting blood pressure (BP) > 130/80->140/85 mmHg), those pregnant women, those with gastrointestinal disease, and those using tobacco products (old and current) and alcoholic beverages were excluded from the study. Those who regularly exercise extensively (3 times a week for >15 min, e.g., by aerobics) were also not included. None of the participants had previously undergone gastrointestinal system surgery; i.e., all participants did not have any medical illness other than being underweight (thin), overweight, or obese. Experiments were made “blindly,” without knowledge of the BMI of the participants in order to reduce bias.

### 2.1. Immunohistochemistry

Immunohistochemical procedures were used as previously described by Hsu and Raine [24]. Sections were taken from paraffin blocks of 4-5 *μ*m thickness. The deparaffinized tissues were passed through a gradual alcohol series and boiled in a microwave (750 W) for 7 + 5 min at pH 6 in citrate buffer solution for antigen retrieval. After boiling, the tissues were incubated for 5 min with hydrogen peroxide block solution (Hydrogen Peroxide Block, TA-125-HP, Lab Vision Corporation, USA) to prevent endogenous peroxidase activity after washing for 3 × 5 min with PBS (Phosphate-Buffered Saline, P4417, Sigma-Aldrich, USA). After washing with PBS for 3 × 5 min, a solution of Ultra V Block (TA-125-UB, Lab Vision Corporation, USA) was applied for 5 min to inhibit background dyeing and incubated at room temperature for 60 min in humidified atmosphere with a 1/200 diluted asprosin primer antibody. The tissues were incubated at room temperature for 30 min in a humidified atmosphere with a secondary antibody (biotinylated Goat Anti-Polyvalent (anti-mouse/rabbit IgG), TP-125-BN, Lab Vision Corporation, USA) after washing with PBS for 3 × 5 min after application of the primer antibody. The tissues were washed with PBS for 3 × 5 min after the application of the secondary antibody and incubated at room temperature for 30 min with Streptavidin Peroxidase (TS-125-HR, Lab Vision Corporation, USA). After the solution of 3-amino-9-ethylcarbazole (AEC) Substrate+AEC Chromogen (AEC Substrate, TA-015 and HAS; AEC Chromogen, TA-002-HAC, Lab Vision Corporation, USA) was added to the tissues and images taken on a light microscope, it was washed with PBS concurrently. Tissues were counterstained with Mayer's hematoxylin before being washed with PBS and distilled water before being mounted with a coverslip (Large Volume Vision Mount, TA-125-UG, Lab Vision Corporation, USA). Slides were photographed and examined with a Leica DM500 microscope (Leica DFC295).

### 2.2. Asprosin Measurements and Test Validations

Asprosin levels in blood and saliva samples of the control group, underweight (weak), overweight, and class I, class II, and class III obese patients were studied with ELISA Kit Shanghai sunredbio (SRB) Technology Co. Ltd, catalog no. 201-12-3287, Shanghai, China. The assay range of the Human Asprosin ELISA kit was 1-300 ng/mL (intra-assay: CV value < 10%, interassay: CV value < 12%); sensitivity was 0.756 ng/mL. Plates were washed with an automatic washer BioTek ELX50 (BioTek Instruments, USA), and absorbance was measured with ChroMate, Microplate Reader P4300 (Awareness Technology Instruments, USA). Asprosin test results were given in ng/mL. A total of 116 biological samples were tested for asprosin, with some samples being measured twice.

The kits used were used to measure the levels of asprosin in blood and other biological samples. Assay validation (linearity, recovery, intra-inter assay CV value) was carried out as previously described by Aydin [25] in order to check the amount of asprosin in saliva samples that were measured as accurately as blood in this study. To show linearity, biological samples were diluted to 1/2, 1/4, and 1/8 before asprosin levels were measured. To remove the recoveries, the basal amount of asprosin in each biological sample was first measured. Then, 10 ng/mL pure asprosin was added to the samples whose basal amounts were known. The recoveries were calculated taking into account the amount of asprosin measured in the samples in cases where there was no interference with them (i.e., was the expected amount of asprosin). In addition, coefficients of variation (CV) were calculated by measuring quantities of asprosin within day (intra-assay) and between day (interassay) samples. CV values were calculated by multiplying the standard deviations of the asprosin values by 100 and dividing by the mean asprosin values. CV values of <15% were taken indicating that this kit accurately quantified salivary asprosin levels.

### 2.3. Statistical Analysis

Statistical evaluation of the data used the Statistical Package for the Social Sciences (SPSS) 22 package program. When the variables were distributed normally, Student's *t*-tests were used to identify statistically significant differences between the 2 groups. When the variables were distributed nonnormally, Mann-Whitney *U* tests were used to compare differences between 2 independent groups. One-way analysis of variance (ANOVA) was used to compare continuous data between groups. The correlation between the groups was assessed by the Spearman correlation test. *p* < 0.05 was taken as statistically significant.

## 3. Results

There was no statistically significant difference between the ages of the participants in any of the groups (Table 1). The control group had a BMI of 21.86 ± 1.96 kg/m^2^, the underweight group had a BMI of 15.5 ± 0.71 kg/m^2^, the overweight group had a BMI of 27.68 ± 1.2 kg/m^2^, the obese group in class I had a BMI of 32.8 ± 1.55 kg/m^2^, the obese group in class II had a BMI of 36.9 ± 1.75 kg/m^2^, and the obese group in class III had a BMI of 45.77 ± 4.35 kg/m^2^ (Table 1). Glucose levels of all the groups were within normal physiological limits. However, there was an increase as BMI increased, but it was not statistically significant. Regarding the lipid profiles of the participants, there was no difference between the control and overweight participants in total cholesterol, but there was an increase in total cholesterol as BMI increased. When the total cholesterol levels of the control and the class III obese groups were compared, there was a statistically significant increase in the class III obese group. In terms of the HDL cholesterol values of the groups, it was 54.32 ± 5.46 mg/dL in the control group, but there was a gradual decrease in the other groups, the highest being in the class III obese group. A gradual increase was observed when LDL cholesterol and TG values were compared with control values. Comparing LDL cholesterol and TG values with control values, these were significantly higher in class III obesity.

There was a negative correlation between blood and saliva asprosin with HDL cholesterol, whereas saliva and blood LDL, TG, BMI, and glucose values were positively correlated (Table 2). Other correlations that were found are summarized in Table 2.

**Figure 1:**
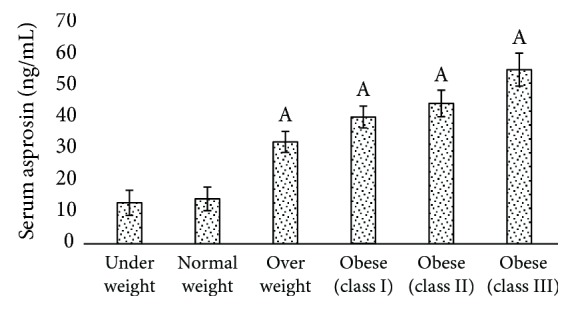
Comparison of blood asprosin values of underweight, normal weight, and overweight individuals and participants with classes I, II, and III obesity. ^a^Normal weight versus another group (*p* < 0.005).

**Figure 2:**
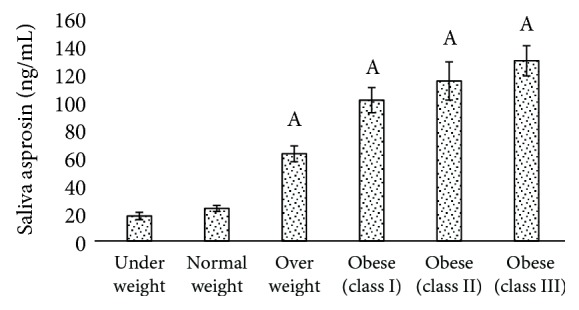
Comparison of salivary asprosin values of underweight, normal weight, and overweight individuals and participants with classes I, II, and III obesity. ^a^Normal weight versus another group (*p* < 0.005).

Asprosin levels of the control group were 14.0 ± 3.75 ng/mL and 23.86 ± 2.22 ng/mL in serum (Figure 1) and saliva (Figure 2), respectively. Minimum asprosin levels were detected in serum of underweight subjects. Blood and salivary asprosin levels gradually increased with increase in BMI (Figures 1 and 2). Comparing the serum asprosin values of the control group with the other groups, increased values were found in overweight subjects by ~2-fold to ~3-fold in classes I and II obesity and ~4-fold in class III obesity. Saliva values of control subjects compared with other groups showed asprosin levels to be ~3-fold higher in overweight, ~4-fold in class I obesity, ~5-fold in class II obesity, and ~6-fold higher in class III.

**Figure 3:**
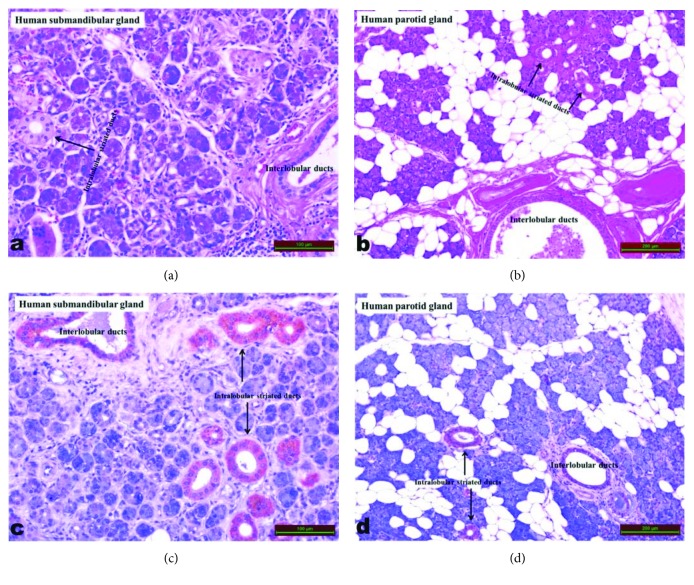
Asprosin immunoreactivity in human submandibular and parotid salivary glands (intralobular striated ducts and interlobular ducts of submandibular and parotid salivary glands). Red-colored areas (arrows) indicate asprosin immunoreactivity.

Submandibular (Figure 3(a)) and parotid (Figure 3(b)) salivary glands were studied for asprosin immunoreactivity. Microscopy showed that asprosin immunoreactivity occurred in intralobular striated ducts and interlobular ducts of submandibular (Figure 3(c)) and parotid (Figure 3(d)) glands. The amount of asprosin in the supernatants of submandibular glands was 14.4 ng/mg tissues whereas the amount of asprosin in the supernatants of parotid glands was 0.88 ng/mg tissue.

## 4. Discussion

Obesity is a serious public health problem that has increasingly high morbidity and mortality in the world, can be seen as a result of genetic, metabolic, or endocrine system disorders, and shortens life span if untreated and impairs quality of life [1, 2]. Adverse changes in lifestyle (high-fat diets) in developed and developing countries are among the major risk factors for obesity, resulting in a poor lipid profile [26]. Comparing lipid profile values with control values, the lowest triglyceride and total cholesterol levels were seen in underweight individuals. On the other hand, HDL cholesterol levels decrease while triglyceride and LDL cholesterol levels increase as the level of obesity increases (obesity classes I, II, and III); i.e., there is a direct correlation between the increase in BMI and the deterioration in the lipid profile. With a unit change in BMI, HDL is known to decrease to 0.69 mg/dL and triglycerides, cholesterol, and LDL to increase in female individuals [27]. Similarly, with a unit change in BMI, HDL decreases, whereas triglycerides, cholesterol, and LDL increase in males. Therefore, the lipid profiles that deteriorate due to obesity in this study are compatible with previous reports. We also recorded a partial increase in glucose levels where there was a statistically significant increase in obesity.

Kara et al. [28] showed that the obesity grade is directly correlated with blood glucose. Another study [29] noted a direct correlation between obesity and glucose. We examined all the details to see any possible link between asprosin amount and BMI; we noted that when BMI increased, the amount of asprosin increased, and BMI decreased in correlation with the amount of asprosin in thin individuals. Plasma asprosin levels seem to be elevated in insulin-resistant animals and humans [16]. With obesity, insulin resistance also increases. Individuals with insulin resistance quickly feel hungry, having a feeling of late saturation [30]. Therefore, there is a direct link between asprosin and obesity. Because asprosin increases appetite by activating agouti-related peptide (AgRP) neurons via cAMP, this increases food intake [15, 17]. In addition, asprosin present in nanomolar levels in the circulation is mainly secreted by white fat tissue [15], which increases with obesity [31]. As this occurs, the white fat tissue increases, and correspondingly, increase occurs in asprosin levels, which in turn results in agouti-related peptide (AgRP) neurons to be activated through cAMP to increase appetite [15]. Food intake increases as appetite increases, which leads to excess uptake, and energy will be converted to fat mass, with obesity continuing to increase within this cycle [31]. The very high amounts of asprosin in morbid obesity that we observed support this mechanism. Asprosin-dependent high glucose production may also increase glucose toxicity; i.e., hyperglycemia itself disrupts the function of pancreatic insulin-secreting beta cells, thereby increasing insulin resistance [32]. Thus, one of the possible factors responsible for insulin resistance in obese and type 2 diabetes patients may be asprosin [32].

Depending on the metabolic effects of diseases such as obesity and diabetes, many associated diseases (e.g., cardiovascular diseases) may have developed in this way. On the other hand, as is well known, glucose production in the liver is mediated by glucogenolysis or gluconeogenesis [33]. To date, the main reason for the increase in hepatic gluconeogenesis may be related with hyperglycemia or an increase of gluconeogenic precursors, such as lactate, alanine, and glycerol. Eventually, fasting hyperglycemia occurs. It has even been assumed that fasting hyperglycemia is entirely due to an increase in liver glucose production [33]. The reason for the increase in glucose production in the liver may be due to asprosin, which is because asprosin increases in starvation and causes the liver to release glucose [19].

We show the novel finding that human salivary glands have the ability to synthesize asprosin. Asprosin immunoreactivity was observed in intralobular striated ducts and interlobular ducts in the submandibular and parotid salivary glands. When the asprosin immunoreactivity of submandibular glands was compared with the asprosin immunoreactivity of parotid glands, the submandibular glands was more reactive and therefore have a greater asprosin production capacity, a finding confirmed with salivary gland tissue supernatants. Submandibular glands had 14.4 ng/mg tissue asprosin whereas parotid glands had 0.88 ng/mg tissue asprosin. The parotid is a serous gland, which is aqueous and rich in fluids, enzymes, and mineral salts, but secretes less organic matter (mucous) [34]. The submandibular gland produces both serous and mucous secretions. This is the secretion that allows the taste to be recognised from any material [35]. Asprosin in the human salivary gland is produced and secreted by acinar cells, as are other salivary proteins [36–38].

Demonstration of asprosin production in the striated ducts of the salivary gland shows compatibility with many previously reported peptide or protein molecules (ghrelin [23], irisin [39], vascular endothelial growth factor [40], dermcidin [41], and others). This means that striated and interlobular ducts of human salivary glands are important endocrine organs of the body that can synthesize a large number of peptide and protein-structured hormones. It is also of interest to note that human saliva contains a very small amount of insulin [42], and the salivary glands seem to channel proteins out from the saliva to the blood circulation [23, 37–41], which was our reason in this study for measuring the amounts of salivary asprosin.

Approximately 99% of saliva is water, 1% being due to large organic molecules (peptides, proteins, glycoproteins, and lipids) and small organic molecules (glucose and urea) as well as electrolytes (sodium, calcium, and phosphate) [35–37]. Saliva is secreted mainly from the major and minor salivary glands. About 69% of the saliva is secreted from the submandibular gland, 26% from the parotid gland, 5% from the sublingual major salivary glands, and 5% from the tonsils, lips, cheeks, palate molars, and retromolar surfaces of many minor glands located in the mucosa [23, 35–41]. We also report for the first time that the saliva contains asprosin hormone. Even when the amount of asprosin in saliva was compared with the amount of asprosin in blood samples taken simultaneously, the levels in the saliva were higher and similar to the blood, and the amount of asprosin increased as BMI increased. Higher amounts of asprosin in saliva than in blood show us that one of the major sources of asprosin is the salivary gland. Asprosin or other peptides, including ghrelin in human saliva, might influence on the proliferation of oral keratinocytes.

Previous studies have reported that salivary gland duct proteins are released into saliva and blood circulation [23, 35–41]. One of the main sources of asprosin in the saliva can be plasma; i.e., asprosin may have passed from plasma to saliva depending on the level of saturation. The other possible source of asprosin in the saliva is the asprosin synthesized in both the plasma and salivary glands. High amounts of asprosin in saliva further strengthen the second possibility. As a result, and regardless of the origin of the asprosin in the saliva, the saliva contains asprosin, which is a novel finding, and there are no other reports for comparison. Salivary asprosin levels were measured here using blood asprosin kits; the Sunred brand kit we used to measure salivary asprosin levels gave us the same precision as for blood asprosin levels. The kit in our work has an intra-assay CV value of 9% and an interassay CV value of 12% in saliva, which confirms that these kits can accurately measure the asprosin value in biological fluids. If asprosin is going to be measured using different branded kits in other biological fluids (for example, urine), including saliva, we recommend validating the assay experimentally in order to check whether the kit accurately measures asprosin. Interlobular ducts (called Pars inisyalis, and streaked ducts are named as intralobular ducts because of their intralobular location) and striated ducts of the parotid and submandibular salivary glands also produce asprosin. Asprosin produced in the human salivary glands may have passed into saliva through Stenon channels from the parotid gland and Wharton channels from the submandibular gland [43], meaning the saliva contains asprosin.

Although the number of participants in this study was limited, and there was no match between individuals in each group, a direct link between asprosin and obesity has been clearly established. It is hoped that future studies with high-involvement subjects will clearly reveal the link between obesity and asprosin.

## 5. Conclusions

In conclusion, our findings overall show that as HDL-C levels decrease, LDL-C and asprosin values increase while BMIs of participants decrease. The novel finding is that asprosin is directly linked to obesity. This also first report asprosin being detected in saliva and the increased asprosin level in saliva correlating with increased BMI. In addition, the salivary glands produce asprosin. If one wishes to investigate the relationship between other metabolic diseases and asprosin, saliva samples can be used instead of blood, especially in children, because salivary glands produce about 1-2 liters of saliva (noninvasive and easy to handle) every day and contain many molecules including asprosin.

## Figures and Tables

**Table 1:** Comparison of age, BMI, glucose, and lipid profiles of the groups.

Group	Age (year) *p* value	BMI (kg/m^2^) *p* value	Glucose (mg/dL) *p* value	Total C (mg/dL) *p* value	HDL-C (mg/dL) *p* value	LDL-C (mg/dL) *p* value	TG (mg/dL) *p* value
Underweight	37.00 ± 4.75 *0.661*	15.50 ± 0.71^∗^ *0.002*	78.00 ± 8.04 *0.234*	169.00 ± 14.14 *0.852*	40.00 ± 3.09^∗^ *0.234*	109.00 ± 11.21 *0.626*	65.00 ± 7.42 *0.379*
Normal weight	34.25 ± 4.79	21.86 ± 1.96	84.84 ± 8.98	177.07 ± 35.11	54.32 ± 5.46	103.36 ± 10.84	110.16 ± 12.89
Overweight	37.95 ± 5.46 *0.166*	27.68 ± 1.20^∗^ *0.001*	87.32 ± 8.31 *0.389*	177.26 ± 34.73 *0.747*	44.89 ± 5.34^∗^ *0.047*	108.16 ± 10.67 *0.648*	135.68 ± 14.54^∗^ *0.043*
Obese class I	35.80 ± 5.66 *0.763*	32.80 ± 1.55^∗^ *0.001*	92.00 ± 10.19^∗^ *0.029*	182.60 ± 32.15 *0.306*	43.60 ± 5.62 *0.090*	111.20 ± 12.95 *0.355*	137.20 ± 1.59 *0.181*
Obese class II	38.38 ± 4.74 *0.402*	36.92 ± 1.75^∗^ *0.001*	97.85 ± 12.48^∗^ *0.001*	190.00 ± 37.88 *0.216*	42.62 ± 7.04^∗^ *0.035*	117.69 ± 12.00 *0.126*	138.92 ± 14.29^∗^ *0.044*
Obese class III	37.05 ± 4.35 *0.544*	45.77 ± 4.35^∗^ *0.001*	101.23 ± 21.03^∗^ *0.001*	195.00 ± 25.28^∗^ *0.007*	38.36 ± 7.51^∗^ *0.001*	128.41 ± 14.37^∗^ *0.004*	145.00 ± 4.02^∗^ *0.002*

BMI: body mass index; HDL-C: high-density lipoprotein cholesterol; LDL-C: low-density lipoprotein cholesterol; TG: triglyceride. Data are shown as means ± SD. *p* value: normal weight group versus other groups. ^∗^Significant *p* value.

**Table 2:** Correlations between biochemical parameters and demographic parameters.

Associated parameters	*r* value	*p* value
Saliva asprosin-serum asprosin	+0.491	0.001
Saliva asprosin-BMI	+0.612	0.001
Saliva asprosin-glucose	+0.224	0.019

Saliva asprosin-total cholesterol	+0.209	0.028
Saliva asprosin-HDL cholesterol	–0.293	0.002
Saliva asprosin-LDL cholesterol	+0.190	0.047

Saliva asprosin-TG	+0.263	0.005
Serum asprosin-BMI	+0.677	0.001
Serum asprosin-glucose	+0.362	0.001

Serum asprosin-HDL cholesterol	–0.344	0.001
Serum asprosin-LDL cholesterol	+0.255	0.007
Age-glucose	+0.391	0.001

Age-total cholesterol	+0.250	0.008
Age-HDL cholesterol	–0.247	0.009
Age-LDL cholesterol	+0.293	0.002

Age-TG	+0.328	0.001
Gender (female)-HDL cholesterol	+0.214	0.024
Gender (female)-LDL cholesterol	–0.206	0.031

Gender (female)-TG	–0.480	0.001
BMI-glucose	+0.525	0.001
BMI-total cholesterol	+0.220	0.021

BMI-HDL cholesterol	–0.434	0.001
BMI-LDL cholesterol	+0.328	0.001
BMI-TG	+0.303	0.001

Glucose-TG	+0.249	0.009
Total cholesterol-LDL cholesterol	+0.618	0.001
Total cholesterol-TG	+0.501	0.001

HDL cholesterol-LDL cholesterol	–0.237	0.013
HDL cholesterol-TG	–0.373	0.001
LDL cholesterol-TG	+0.447	0.001

BMI: body mass index; HDL-C: high-density lipoprotein cholesterol; LDL-C: low-density lipoprotein cholesterol; TG: triglyceride.

## Data Availability

The data used to support the findings of this study are available from the corresponding author upon request.

## Additional Points

*Highlights.* (1) Salivary glands show asprosin immunoreactivity. (2) Immunoreactivity in salivary glands was observed in interlobular and striated ducts. (3) Saliva contains asprosin. (4) The lowest asprosin was detected in the underweight participants, and the highest asprosin was detected in the class III obese participants. (5) Saliva and blood asprosin were correlated with each other. (6) There were correlations between lipid profiles and the amounts of asprosin.
